# Boron Nanosheet-Supported Rh Catalysts for Hydrogen Evolution: A New Territory for the Strong Metal-Support Interaction Effect

**DOI:** 10.1007/s40820-021-00662-y

**Published:** 2021-06-08

**Authors:** Keng Chen, Zeming Wang, Liang Wang, Xiuzhen Wu, Bingjie Hu, Zheng Liu, Minghong Wu

**Affiliations:** 1 Institute of Nanochemistry and Nanobiology, School of Environmental and Chemical Engineering, Shanghai University, 99 Shangda Road, BaoShan District, Shanghai, 200444 People’s Republic of China; 2 School of Materials Science and Engineering, Nanyang Technological University, 50 Nanyang Avenue, Singapore, 639798 Singapore; 3 Shanghai Applied Radiation Institute, Shanghai University, 333 Nanchen Road, Baoshan District, Shanghai, 200444 People’s Republic of China; 4 Key Laboratory of Organic Compound Pollution Control Engineering (MOE), Shanghai University, Shanghai, 200444 People’s Republic of China

**Keywords:** Boron nanosheets, Dispersive rhodium nanoparticles, Electrocatalysis, Hydrogen evolution reaction, Strong metal-supported interaction

## Abstract

High-efficiency electrochemical hydrogen evolution reaction (HER) offers a promising strategy to address energy and environmental crisis. Platinum is the most effective electrocatalyst for the HER. However, challenging scarcity, valuableness, and poor electrochemical stability still hinder its wide application. Here, we designed an outstanding HER electrocatalyst, highly dispersed rhodium (Rh) nanoparticles with an average diameter of only 3 nm supported on boron (B) nanosheets. The HER catalytic activity is even comparable to that of commercial platinum catalysts, with an overpotential of only 66 mV in 0.5 M H_2_SO_4_ and 101 mV in 1 M KOH to reach the current density of 10 mA cm^−2^. Meanwhile, the catalyst exhibited impressive electrochemical durability during long-term electrochemical processes in acidic and alkaline media, even the simulated seawater environment. Theoretical calculations unraveled that the structure–activity relationship between B(104) crystal plane and Rh(111) crystal plane is beneficial to the release of hydrogen, and surface O plays a vital role in the catalysis process. Our work may gain insights into the development of supported metal catalysts with robust catalytic performance through precise engineering of the strong metal-supported interaction effect.

**Supplementary Information:**

The online version contains supplementary material available at 10.1007/s40820-021-00662-y.

## Highlights

- Ultra-small and highly dispersed rhodium nanoparticles anchored in 2D ultra-thin boron nanosheets (BNS) were synthesized by a rapid NaBH_4_ reduction and facile freeze-dry approach.
- Due to the strong metal-support interaction effect, the optimized electrocatalyst exhibits excellent hydrogen evolution reaction (HER) catalytic activity and stability in the wide pH range electrolytes.
- Based on the stable surface, the oxidized boron surface facilitates the coupling of BNS and metal, giving catalyst outstanding HER performance.

**Supplementary Information:**

The online version contains supplementary material available at 10.1007/s40820-021-00662-y.

## Graphical abstract

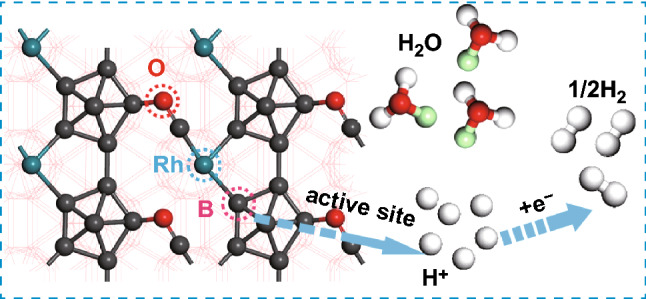

## Introduction

The urgency of addressing the global traditional energy crisis and environmental pollution has given impetus to worldwide research in economically sustainable, highly efficient, and clean energy technologies [1, 2]. Hydrogen, a clean, renewable, and abundant source, has been deemed a potential candidate for substituting fossil fuels [3]. Among the numerous hydrogen evolution reaction (HER) technologies, highly efficient water electrolysis has been considered as an ideal approach to resolving the looming energy and environmental crisis [4]. Thus far, platinum (Pt) is still the most superior electrocatalyst for HER with low overpotentials, high exchange current densities, and small Tafel slopes [5–7]. However, the scarcity, valuableness, and poor electrochemical stability of Pt seriously impede its development for the industrial production of hydrogen [6]. Given this, there is still a severe challenge to explore earth abundance, highly efficient, and long-term stable electrocatalysts for HER.

Supported metal catalysts (SMCs) have always been one of the hot spots in the field of heterogeneous catalysis because the strong metal-supported interaction (SMSI) endows SMCs higher catalytic activity and better stability than the pure metal nanoparticle catalyst [8, 9]. For SMCs, most supports not only play the single role of physical carriers, but also interact with the active metal. Among all the interactions, the so-called SMSI effect has been mainly applied in oxide-supported metal catalysts and opens a new era in the field of heterogeneous catalysis [10, 11]. Nevertheless, due to the low coordination, metal atoms have extremely high surface free energy and tend to agglomerate during synthesis or under working conditions, being unable to achieve the theoretical atom utilization efficiency [12, 13]. Besides, conventional supports of SMCs such as metals and their derivatives (i.e., oxides, nitrides, carbides, and sulfides) are bulk, with less exposed active sites, which further hinders the achievement of theoretical catalytic efficiency. Moreover, the instability of these metal-based substrates in a harsh electrolytic environment (strong acid and alkali) similarly forces us to search for alternatives [3, 14]. Although support like porous carbon exhibits a large specific surface area, it cannot achieve complete participation of metal active sites in the reaction [15]. Therefore, it is crucial to identify a metal-free substrate to achieve high catalytic efficiency and fantastic durability of SMCs.

Two-dimensional (2D) materials demonstrate rapid mass/electron transfer capabilities, twice catalyst area, and exceptional stability, impelling them superior candidates for supports of SMCs [16, 17]. Noteworthy, the thin layer 2D materials exhibit almost twice the catalyst area, which can promote the fully expose the active sites and maximum atom utilization efficiency, thereby improving the intrinsic properties of the catalyst. Boron (B) has received extensive attention due to its earth abundance and impressive physicochemical property [18, 19]. Beyond the graphene materials, the 2D thin boron nanosheets (BNS) have been demonstrated to be a novel and promising alternative to 2D material, due to their fascinating 2D graphene-like structure, tunable bandgap, high carrier mobility, and superconductivity [20–23]. Theoretical investigations confirmed that BNS coupled metals exhibit robust catalytic activity because of their unique electron-deficient features, highlighting the value of BNS as a substrate for the design of SMCs [19, 24, 25]. Inspired by these studies, we raise an interesting hypothesis: will SMCs supported by BNS ever rival commercial Pt/C in HER electrocatalysis?

Herein, we designed a novel sonication-assisted liquid-phase exfoliation method to prepare high-quality 2D BNS and a rapid chemical reduction method to synthesize highly dispersed small-size rhodium nanoparticles anchored on BNS (Rh NP@BNS). Rh NP@BNS exhibited outstanding performance and long-term stability for the HER in both acidic and alkaline solutions, even the simulated seawater environment. Theoretical calculation further studied the structure–activity relationship between material and properties. Our work may unlock a groundbreaking avenue for the green preparation of future 2D electrocatalytic HER materials and provide new insights for the design of SMC with a high catalytic activity using the SMSI effect as a guide.

## Experimental Section

### Materials

Commercial boron powder (99.9%) was purchased from Aladdin. Rhodium chloride (RhCl_3_·3H_2_O, 98%+) was purchased from Adamas. Ethanol (Et-OH, AR), *N*-methyl-2-pyrrolidone (NMP, AR), sodium borohydride (NaBH_4_, AR), sulfuric acid (H_2_SO_4_, AR), potassium hydroxide (KOH, AR), sodium chloride(NaCl, AR) and isopropanol (AR) were purchased from Shanghai Titan Technology Co. Ltd. Nafion solution and commercial Pt/C were purchased from Alfa Aesar. All chemicals were used without any further purification.

### Synthesis of BNS

BNS was prepared by sonication-assisted liquid-phase exfoliation technologies. Firstly, the commercial boron powder with the initial concentration of 2.0 mg mL^−1^ was dispersed in the mixed NMP and Et-OH (1:1 v/v) and sealed with a preservative film. Secondly, the solution was then sonicated for 8 h in an ice bath and centrifuged at 1,000 rpm for 15 min to discard the large chunks of bulk B. Thirdly, the supernatant was then centrifuged at 18,000 rpm for 15 min and washed three times using Et-OH. The residue Et-OH was removed after by vacuum rotary evaporation. At last, the precipitates were collected for future use after dried at room temperature.

### Preparation of Rh NP@BNS

BNS (20 mg) and 380 µL of RhCl_3_·3H_2_O solution (10 mmol L^−1^) were dissolved in 30 mL of deionized water and then sonicated until the solution became well-distributed. Afterward, 75 mg NaBH_4_ was added into the solution and the ultrasonic continued for 2 h. The mixed solution was then centrifuged at 18,000 rpm for 15 min and washed three times using deionized water. Subsequently, the solution was then freeze-dried for 48 h to obtain the solid product. The obtained powder was collected for future use. The samples with different Rh content were synthesized by changing the volume of RhCl_3_·3H_2_O solution. The only difference between the preparation of Rh NP and Rh NP@BNS is that no BNS was added.

### Electrochemical Measurements

All the electrochemical measurements were performed in a three-electrode system on an electrochemical workstation (CHI760e, Shanghai Chenhua Instruments Co., China) at room temperature. Taking the Rh NP@BNS electrode as an example, the catalyst ink was prepared by dispersing electrocatalysts (5.0 mg) in a mixture of 700 µL deionized water, 250 µL isopropanol, and 50 µL of 5% Nafion solution. The mixture was sonicated in an ice bath for 1 h. Then, the aqueous dispersion of the catalyst (10 µL, 5.0 mg mL^−1^) was completely dropped to the glassy carbon electrode. The mass loading of the electrode is 0.254 mg cm^−2^. A rotating disk electrode (RDE) was applied for HER performance testing in a three-electrode cell configuration. A saturated Hg/Hg_2_Cl_2_ electrode (acid electrolyte) or an Ag/AgCl (in 3 M KCl solution) electrode (alkaline electrolyte) as the reference electrode, a graphite rod as the counter electrode, and the glassy carbon electrode supported catalysts as the working electrode. All potentials were calibrated to the RHE by the equation *E*_RHE_ = *E* (Hg/Hg_2_Cl_2_) + 0.059 pH + 0.244 and *E*_RHE_ = *E* (Ag/AgCl) + 0.059 pH + 0.200.

The HER performance was measured in N_2_ saturated aqueous 0.5 M H_2_SO_4_ (pH = 0.3), 1.0 M KOH (pH = 14), and 1.0 M NaCl (pH = 7) at a scan rate of 50 mV s^−1^ with 100 cyclic voltammetry (CV) cycles in the range of 0.3 to − 0.8 V_RHE_. Linear sweep voltammetry (LSV) with a scan rate of 10 mV s^−1^ at 1600 rpm after 100 CV cycles in the range of 0.3 to − 0.8 V_RHE_. Tafel curves were then obtained from linear sweep voltammograms using a scan rate of 10 mV s^−1^. The electrochemical impedance spectroscopy (EIS) was executed in the frequency range of 100 kHz to 1 mHz with a modulation amplitude of 10 mV.

Cycling stability tests. The LSV curves were recorded for the first cycle and after 1000 CV sweeps between 0.3 and − 0.8 V_RHE_ at 50 mV s^−1^. All the LSV curves were performed in the different electrolytes at a scan rate of 10 mV s^−1^.

Durability measurements. The chronoamperometry test of Rh NP@BNS was obtained by running the test for 20 h under a constant overpotential corresponding to the current density of 10 mA cm^−2^ (η10).

### Models and Computational Methods of Density Functional Theory (DFT) Calculations

All DFT calculations were carried out using VASP (5.4.4) code with GGA-RPBE exchange–correlation functional [26–28]. The projector augmented wave (PAW) pseudopotential for describing the core-valence interactions and a plane-wave basis set with a kinetic energy cutoff of 450 eV were used [29, 30]. The Brillouin zone in reciprocal space was sampled by Monkhorst–Pack scheme with 4 × 5 × 1 k-points grids for geometry optimization [31]. The convergence threshold was set to 10^–4^ eV in electronic relaxation and 0.02 eV Å^−1^ in Hellmann–Feynman force on each atom. Fermi smearing of *k*_B_*T* = 0.1 eV was employed to speed up the convergence. The ab initio molecular dynamics (AIMD) simulations were performed within the NVT ensemble at *T* = 500 K with a time step of Δ*t* = 3 fs and an overall time scale of 9 ps. The optimized lattice parameter for bulk B was calculated to be 4.91 Å, agreeing well with the experimental result (4.93 Å). (Rhombohedral crystal structure of compounds containing boron-rich icosahedra) A vacuum region in the *z*-direction is set to 15 Å and the surface is periodic in the *x*- and *y*-direction. The size of a chosen supercell is sufficient to avoid the interactions between the imaging cells.

Gibbs free energy of reaction for the elementary steps involving (H^+^  + e^−^) pair transfer was calculated using computational hydrogen electrode (CHE) model [32]. The free energy change (at pH = 0) for each key elementary step was estimated as:

1 $$\Delta G_{n} {(}U{) } = \, \Delta E_{n} + \, \Delta {\text{ZPE }} - \, T\Delta S \, + {\text{ neU}}$$

where ∆*E*_*n*_ is DFT-calculated reaction energy in a vacuum, ∆ZPE is zero-point energy (ZPE) correction based on the calculated vibrational frequencies, *T*∆*S* is the entropy contributions to the reaction at *T* = 298.15 K. VASPKIT code was used to process the calculation results of VASP.

### Characterization

The morphology of materials was observed by scanning transmission electron microscopy (SEM, JEOL JSM-6490). Transmission electron microscopy (TEM) measurements were performed on a JEOL JEM-2100F electron microscope operating at 200 kV. The atomic force microscope (AFM) images were characterized using Bruker Dimension Icon AFM. An X-ray powder diffraction (XRD) pattern was obtained with Rigaku D/max-2500 using CuKa radiation. The functional groups on the sample surface were measured by X-ray photoelectron spectroscopy (XPS, Thermo Fischer, ESCALAB250Xi).

## Results and Discussion

### Characterization of Materials

**Fig. 1:**
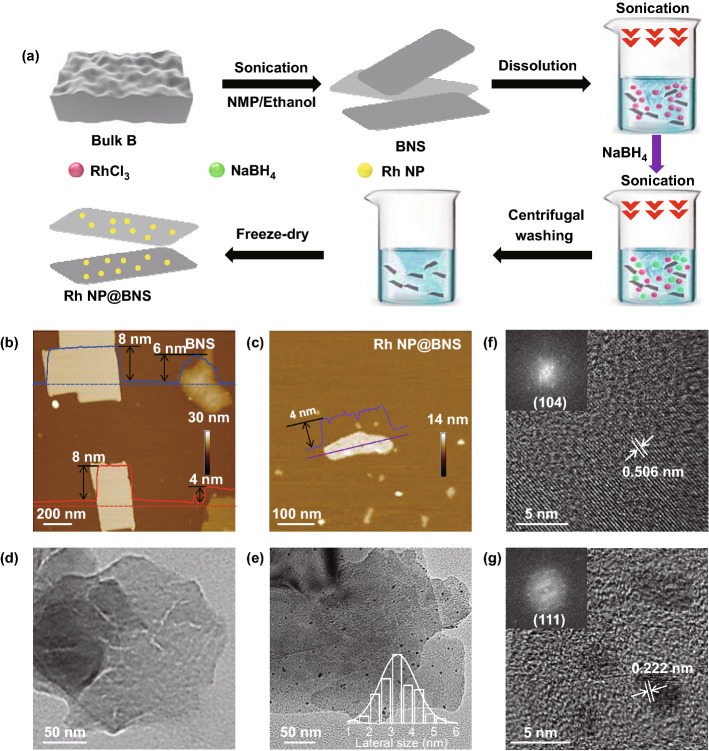
**a** Schematic illustration of the preparation of 2D BNS and Rh NP@BNS. **b, c** AFM images, **d**, **e** TEM images (inset: lateral size distribution of Rh NP), **f**, **g** high-resolution TEM images of Rh NP@BNS (inset: the corresponding FFT patterns)

The typical Rh NP@BNS was prepared via a rapid chemical reduction and facile freeze-dry approach, as schematically shown in Fig. 1a. The BNS was prepared from bulk B by sonication-assisted liquid-phase exfoliation processes. So far, compared to the bottom-up method (e.g., chemical vapor deposition and molecular-beam epitaxy), sonication-assisted liquid-phase exfoliation is a more promising option for mass production of 2D BNS because it does not require harsh conditions. A mixture of NMP and Et-OH (1:1 v/v) was chosen as the liquid exfoliating solvent of bulk B powder. To tailor the controllable thickness and size of the exfoliated B sheets, BNS was obtained by selective centrifugation. In this way, the conversion rate from bulk B to BNS can reach up to 5%, which has the potential for mass production. Subsequently, the loading of Rh NP on BNS involved the addition of the appropriate volume of RhCl_3_·3H_2_O solution to a previously sonicated suspension of BNS solution, followed by rapid reduction with NaBH_4_. Finally, the centrifugal treated sample solution was then freeze-dried to obtain the Rh NP@BNS powder materials.

As shown in Fig. 1b, d, the sheet structure of B was confirmed by AFM and TEM. The thickness of BNS (further presented in Fig. S1a, c, d) was 4–8 nm, and the lateral size of BNS was range from 150 to 250 nm after liquid exfoliation. Compared with the TEM image of bulk B (Fig. S2a), the obvious reduction in the thickness and lateral size of the BNS after exfoliating. Additionally, it can be seen from the SEM images (Fig. S3a, b) that the bulk B with a lateral size of several micrometers exfoliated into several hundred nanometers of B sheets, which further indicates the successful synthesis of the BNS structure. The decreases of thickness and lateral size may ascribe the physical adsorbed and the fragmentary covalent bond grid among B sheets was broken by sonication in exfoliating solution [33]. After Rh NP anchored on the BNS, the Rh NP@BNS still possessed 2D planar-shaped structures. From the AFM (Figs. 1c and S1b, e) and TEM images (Fig. 1e), the unique morphology of Rh NP@BNS is revealed. The Rh NPs (size distribution: 1 to 5 nm, average diameter only 3 nm) are homogeneously distributed within the BNS framework, which is also mutually confirmed in Fig. S2b. The crystal structure of Rh NP@BNS is more clearly visible in the high-resolution TEM image (HRTEM) and corresponding fast-Fourier transform (FFT) pattern. The representative HRTEM (Fig. 1f) confirms the crystalline nature of B sheets, displaying a clear interference fringe with a *d*-spacing of 0.506 nm, corresponding to the (104) plane of a β-rhombohedral B structure. The spacing of the crystal lattices is 0.222 nm in Fig. 1g, which can be attributed to the face-centered cubic (fcc) Rh(111) crystal plane. It should be emphasized that BNS can monodisperse Rh NP with an average particle size of 3 nm, and no agglomeration of Rh NP is observed. Such a 2D planar architecture could contribute to the exposure of more active sites for these nanosheet systems.

**Fig. 2:**
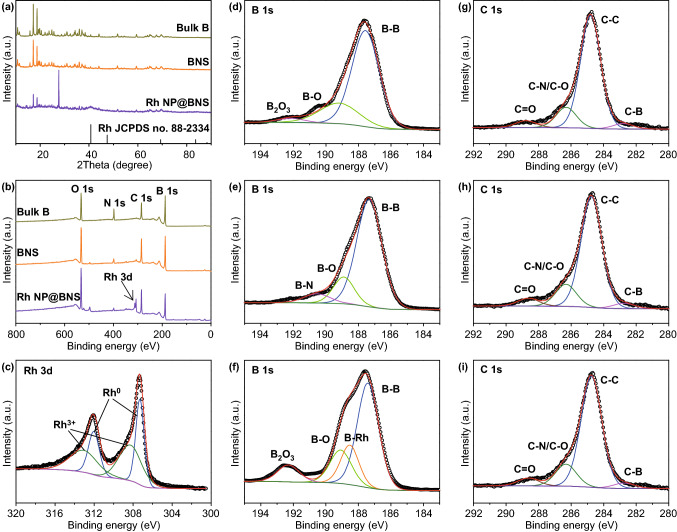
**a** XRD patterns and **b** XPS survey spectra of bulk B, BNS, and Rh NP@BNS. **c** High-resolution spectra of Rh 3d of Rh NP@BNS. **d**-**f** High-resolution spectra of B 1*s* and **g**-**i** C 1*s* of bulk B, BNS, and Rh NP@BNS

XRD patterns were utilized to examine the phase structures of bulk B, BNS, and Rh NP@BNS. As shown in Fig. 2a, the diffraction peaks of bulk B can be attributed to β-rhombohedral B (JCPDS No. 71-0157), while the highest peak at 17.5° belongs to the (104) plane of B (Fig. S4a). The characteristic peaks of BNS are consistent with bulk B, corroborating that BNS retains the crystallinity of the original bulk B. It is worth noting that the characteristic peak of B_2_O_3_ (JCPDS No. 06-0297) (Fig. S4b) appeared at about 27°, which is due to the oxidation of B. The indicated peaks at 41° observed for Rh NP@BNS could be ascribed to the corresponding (111) reflection of the fcc Rh structure (JCPDS No. 88-2334) (Fig. S4c). X-ray photoelectron spectroscopy (XPS) was employed to further analyze the chemical banding state and surface composition of bulk B, BNS, and Rh NP@BNS in Fig. 2b–i. Except for peak B, peaks corresponding to C, O, and N were also detected for all three samples in their XPS survey spectra. The typical SEM images of bulk B (Fig. S5), BNS (Fig. S6), Rh NP@BNS (Fig. S7) as well as the corresponding element mapping images indicated that all samples are mainly composed of B elements, with a small amount of C, N, and O impurities. The existence of C, O, and N impurities is possibly due to the surface contamination that occurs during exposure to the exfoliating solvent and the air atmosphere [34]. The peak centered at 187.7 eV in high-resolution B 1 s spectra corresponds to the B−B bond of bulk B. Moreover, the peaks at 189.1 and 192.2 eV are mainly attributed to the oxidation of B. The peak located at 189.1 eV can be ascribed to the B−O bond in a boron-rich oxide, while the small peak centered at 192.2 eV can be assigned to the formation of B_2_O_3_. It worth noting that the binding energy (BE) peaks of the B−B bond for BNS and Rh NP@BNS are slightly shifted from 187.7 to 187.4 eV, compared to that of bulk B. This phenomenon agrees with some reported 2D BNS [34–36]. Moreover, the small peak corresponding to B_2_O_3_ disappeared for BNS after liquid exfoliation due to interactions between the B sheets and the exfoliating solvent, while another component centered at 190.4 eV was found, possibly attributing to the formation of B–N bonds because of the air contamination and solvent residue shell [34]. Interestingly, compared to BNS, the peak at 192.3 eV corresponding to B_2_O_3_ reappeared for Rh NP@BNS, possibly owing to the destruction of a solvent residue shell on the B sheet surface after underwent NaBH_4_ reduction and freeze-drying processes that provide favorable conditions for air oxidation, which is consistent with the XRD result of Rh NP@BNS. It should be emphasized that the component at 188.5 eV may arise from the formation of a B–Rh bond due to the SMSI effect. Encapsulation of Rh NP anchored on BNS by a thin layer of support-derived boron oxide, which is a well-known characteristic of the classical SMSI effect observed in oxide-SMCs [10, 37]. The Rh 3*d* spectrum in Fig. 2c can be deconvoluted into two types of Rh, the peaks at 307.3 eV (3*d*^5/2^) and 312.0 eV (3*d*^3/2^) belong to Rh^0^ and the other two peaks at 308.3 eV (3*d*^5/2^) and 313.0 eV (3*d*^3/2^) should be vested in Rh^3+^. Figure 2g–i illustrated the high-resolution C 1*s* spectra of bulk B, BNS, and Rh NP@BNS, respectively. The four components near 288.8, 286.3, 284.8, and 282.8 eV can be fitted in all the C 1*s* spectra, which can be allocated to the C–O, C–N/C–O, C–C, and C–B bonds, respectively.

### Electrochemical Performance of Rh NP@BNS

**Fig. 3:**
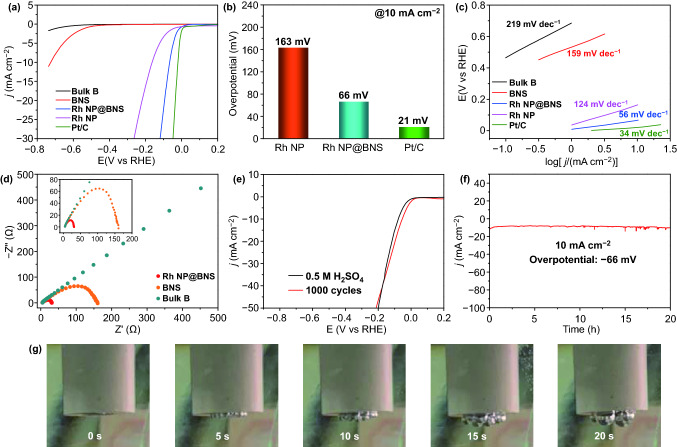
**a** LSV curves of bulk B, BNS, Rh NP@BNS, Rh NP, and Pt/C were performed in 0.5 M H_2_SO_4_ electrolyte. **b** Comparison of the overpotentials at 10 mA cm^−^^2^. **c** The corresponding Tafel slopes were derived from LSV curves. **d** Nyquist plots of bulk B, BNS, and Rh NP@BNS. **e** LSV curves were measured before and after 1000 cycles at 0.2 to – 0.9 V versus RHE in 0.5 M H_2_SO_4_ electrolyte. **f** Time dependence of current density at 66 mV versus RHE. **g** Photos of a working electrode using Rh NP@BNS as a catalyst during CV measurement from 0 to − 0.2 V in 0.5 M H_2_SO_4_ electrolyte. The photos were taken at 5 s intervals

The HER activities of electrocatalysts were evaluated in 0.5 M H_2_SO_4_ aqueous solutions saturated with N_2_ using a three-electrode electrochemical cell. To examine the effect of Rh doping content on the HER activity, we prepared the same catalyst with different Rh doping amount (i.e., *x*-Rh NP@BNS, *x* = 0, 0.5, 1.0, 2.0, 3.0, 5.0, 7.0) under identical experimental conditions using RhCl_3_·3H_2_O as the Rh source for the control experiment. All *x*-Rh NP@BNS samples possessed nonzero cathodic currents (Fig. S8), manifesting conspicuous HER activity. Remarkably, when the Rh doping amount is 0–5.0 wt%, the current density obtained by the catalyst gradually increases, while the current density decreases instead if the doping amount increased to 7.0 wt%. It indirectly proves that the appropriate doping amount of Rh can affect the catalytic performance, and the optimum doping amount is 5.0 wt%. Consequently, 5-Rh NP@BNS was selected for further study. The truly Rh deposited content of 5-Rh NP@BNS was 1.11 wt% (Table S1) obtained by inductively coupled plasma optical emission spectrometry (ICP-OES). For comparison, the electrocatalytic activities of bulk B, BNS, Rh NP, and commercial Pt/C were also measured under the same conditions. As observed in Fig. 3a, b, the current density of 10 mA cm^−^^2^ (η10), a significant parameter of the HER activity, can be identified at − 725 mV for BNS, − 163 mV for Rh NP, − 66 mV for Rh NP@BNS, and − 21 mV for commercial Pt/C. Significantly, the η10 of Rh NP@BNS can even rival commercial Pt/C. As expected, bulk B and BNS exhibit low HER activity, suggested that the introduction of Rh NP could significantly improve the HER activity of BNS and mightiness interaction between BNS and Rh NP. Consistent behaviors can be observed in the Tafel plots in Fig. 3c, where the Tafel slope is 219 mV dec^−1^ for bulk B, 159 mV dec^−1^ for BNS, 124 mV dec^−1^ for Rh NP, and 56 mV dec^−1^ for Rh NP@BNS, in comparison with 34 mV dec^−1^ for Pt/C.

The electrochemical impedance spectroscopy (EIS) was used to examine the charge-transfer kinetics of catalyst-decorated electrodes during the HER process. From the Nyquist plots acquired at − 100 mV vs reversible hydrogen electrode (RHE) (Fig. 3d), we can observe BNS and Rh NP@BNS performed a quasi-semicircle, where the diameter represents the corresponding charge-transfer resistance (*R*_ct_). The *R*_ct_ experienced a decrease for Rh NP@BNS (31 Ω) compared with bulk B (> 500 Ω) and BNS (160 Ω), suggesting that the introduction of Rh NP can improve the electron transfer properties. In addition to its excellent HER catalytic activity, Rh NP@BNS is also a stable and durable catalyst. After 1,000 CV cycles, the LSV curve of Rh NP@BNS did not perform any negative shift in comparison with the initial one, instead, it shifted positively approximately 20 mV at η10. This is evidence for superior electrochemical stability for long-term electrochemical processes [38]. The chronoamperometry test was conducted to assess the durability of the HER catalyst. The result (Fig. 3f) manifested that the Rh NP@BNS possesses long-term operational stability beyond 20 h at 66 mV versus RHE with the current density well-maintained, suggesting that Rh NP is firmly integrated into the BNS backbone and highly stable under electrolytic conditions. The quick emigration of the hydrogen bubbles also confirms the fast kinetics of heterogeneous electron transfer in the Rh NP@BNS catalyst (Fig. 3g). It is worth mentioning that such outstanding HER overpotential and Tafel slope of Rh NP@BNS is superior to many Rh-based HER catalysts reported to date in acidic media (Table S4).

**Fig. 4:**
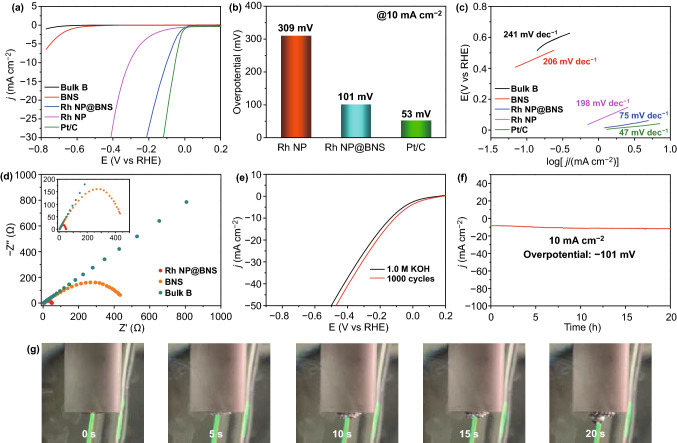
**a** LSV curves of bulk B, BNS, Rh NP@BNS, Rh NP, and Pt/C performed in 1.0 M KOH electrolyte. **b** Comparison of the overpotentials at 10 mA cm^−^^2^. **c** The corresponding Tafel slopes were derived from LSV curves. **d** Nyquist plots of bulk B, BNS, and Rh NP@BNS. **e** LSV curves were measured before and after 1000 cycles at 0.2 to – 0.9 V versus RHE in 1.0 M KOH electrolyte. **f** Time dependence of current density at 66 mV versus RHE. **g** Photos of a working electrode using Rh NP@BNS as a catalyst during CV measurement from 0 to − 0.2 V in 1.0 M KOH electrolyte. The photos were taken at 5 s intervals

The HER activities of 5-Rh NP@BNS were further estimated under N_2_-saturated 1.0 M KOH aqueous solutions. In all x-Rh NP@BNS samples, 5-Rh NP@BNS also displayed the best HER catalytic activity (Fig. S9). The Rh NP@BNS exhibits much superior activity in 1.0 M KOH with the pretty low overpotentials (101 mV) to produce current densities of η10 compared with bulk B (not reached η10), BNS (not reached η10) and Rh NP (309 mV) in Fig. 4a, b. In particular, the needed overpotential to deliver current densities of η10 was 53 mV for commercial Pt/C, which is only 47 mV higher than that of Rh NP@BNS. This is very impressive and can be compared with the latest Rh-based materials reported recently for electrocatalysis HER in alkaline media (Table S5). The corresponding Tafel plots (Fig. 4c) illustrate that Rh NP doping decreases the Tafel slope from 206 to 75 mV dec^–1^ (241 mV dec^–1^ for bulk B, 206 mV dec^–1^ for BNS, 198 mV dec^–1^ for Rh NP, 75 mV dec^–1^ for Rh NP@BNS), slightly larger than that of Pt/C (47 mV dec^–1^), suggesting the Volmer–Tafel mechanism as the HER pathway, in which recombination of chemisorbed hydrogen atoms is the rate-limiting step. In other words, the Rh doping makes access to the electrons and the formation of H_ads_ easier at the interface. The Nyquist plots (Fig. 4d) display a large reduction in the Rct from > 1000 Ω for bulk B, 433 Ω for BNS to 50 Ω for Rh NP@BNS, verifying the exceptional ability of our catalyst to promote electron transport. The robust catalytic stability and durability of the Rh NP@BNS in an alkaline electrolyte are further demonstrated by the LSV curve of CV cycles (Fig. 4e) and chronoamperometry test (Fig. 4f). The rapid precipitation of hydrogen bubbles in 20 s further confirms the strong activity of the Rh NP@BNS catalyst in the alkaline electrolyte (Fig. 4g).

Considering that Rh NP@BNS has superior electrocatalytic activity in acidic and alkaline electrolytes, we further investigated them in neutral NaCl electrolyte to simulate the HER performance in the seawater environment. As summarized in Fig. S10, it took Rh NP@BNS an overpotential of 489 mV to reach a current density of η10 in the 1.0 M NaCl electrolyte. Theoretical calculations indicated that the electrocatalyst may require a kinetic overpotential of up to 480 mV for water oxidation without generating any hypochlorite (Cl^−^  + 2OH^−^  = ClO^−^  + H_2_O + 2e^−^) [39, 40]. Therefore, it is not surprising that Rh NP@BNS requires 489 mV of overpotential to reach a current density of η10 in 1.0 M NaCl electrolyte. On the contrary, it is more likely to become a suitable electrocatalyst for seawater splitting. Specifically, Rh NP@BNS only needed to have overpotentials of 114 and 137 mV, in 0.5 M H_2_SO_4_ + 1.0 M NaCl and 1.0 M KOH + 1.0 M NaCl electrolytes as the HER electrocatalyst to drive a current density of η10. It can be seen from Fig. S11 that after 10 h of chronoamperometry test in 1.0 M NaCl electrolyte, the current density virtually no changes, demonstrating our electrocatalyst has the potential to provide hydrogen energy for ocean navigation.

### DFT Theoretical Calculation

**Fig. 5:**
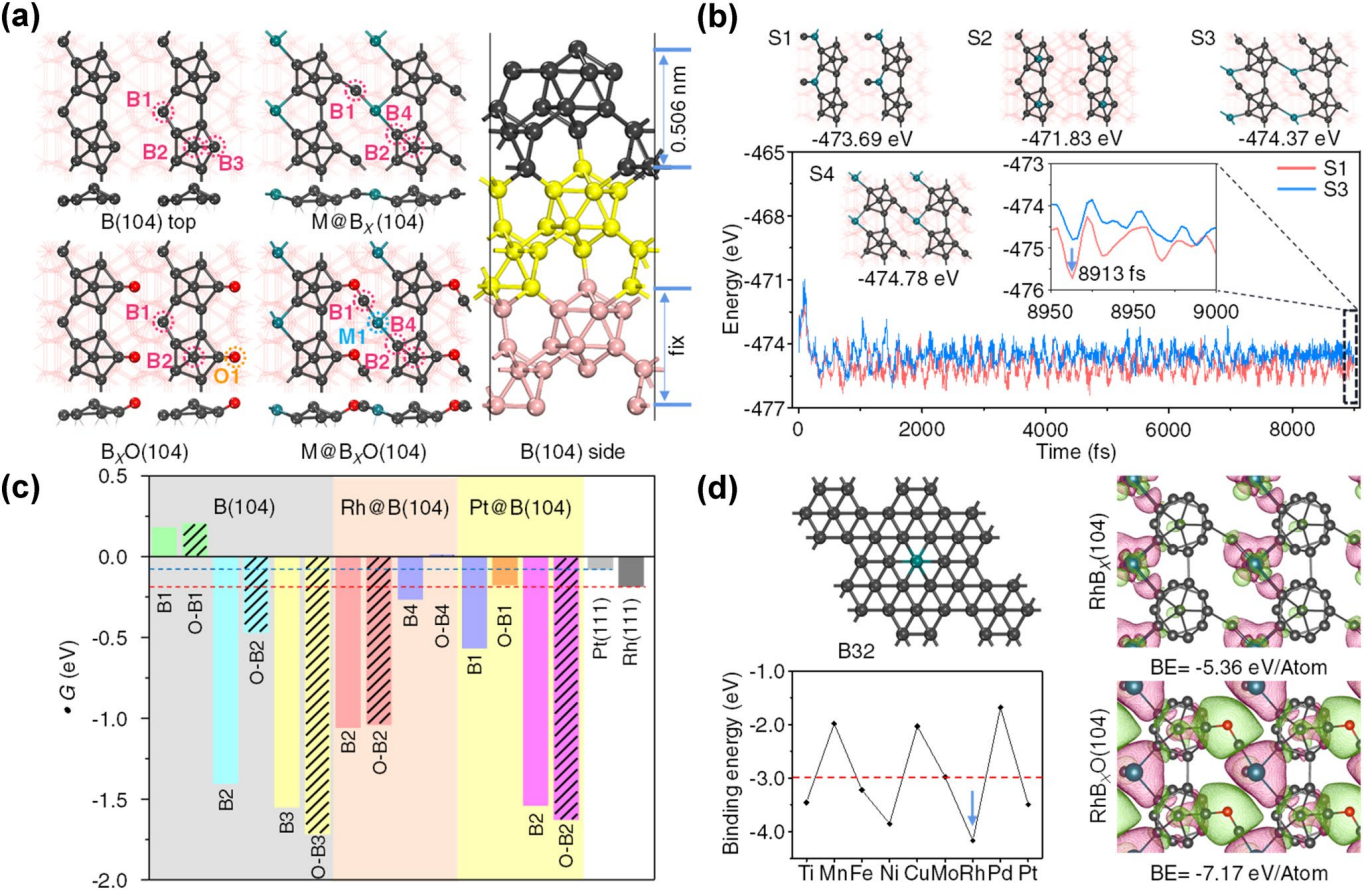
**a** Top and side views of optimized B(104), B_*x*_O(104), M@B_*x*_O(104) geometries structure (*x* = 72, M = Rh, Pt) in a *p*(2 × 2) supercell. **b** Static optimization structures of S1–S3 and the local-minimum dynamics structure S4. Energy progress with time in AIMD simulations at 500 K, the inset shows a snapshot of 8900–9000 fs. **c** ΔG_H*_ of the reaction of H^+^  + e^−^ → H^*^ on the B(104), M@B_*x*_O(104) surfaces and different atom sites, and diagonal lines represent their corresponding oxidized surfaces, respectively. **d** BE of various transition metals (including Ti, Mn, Fe, Ni, Cu, Mo, Rh, Pd, Pt) anchored on B_*y*_(*y* = 32) surfaces. The red dotted line indicates the average BE value. Isosurface of charge density difference (Δ*ρ*) for Rh@B(104), Rh@B_*x*_O(104), Red: charge accumulation; green: charge depletion. Isosurface level = 0.004 e/Bohr^3^. Δ*ρ* = *ρ*(slab + ads) − *ρ*(slab) − *ρ*(ads)

To clarify the structure–activity relationship of Rh NP@BNS, DFT calculations on their active sites and the corresponding electronic structure properties were performed to gain a further grasp into the HER mechanism at an atomic scale. Based on the result of HRTEM images, the B(104) surface was considered as the lowest surface energy. The calculated crystal plane spacing of the model is 5.055 Å, which corresponding to the experimental result (0.506 nm). In addition, the XPS survey spectra elucidated that BNS is partially oxidized to form the B–O bond in the experimental synthesis process. Thus, the periodic *p*(1 × 1) three-layer slabs of B(104), B_*x*_O(104), M@B_*x*_(104), M@B_*x*_O(104) were employed to accommodate key adsorbates H* involved in HER (Fig. 5a). The bottom one layer was fixed while the top two layers and the adsorbates relax. Previous work about N_2_ adsorption confirmed that the relaxation of two layers thickness of B(104) is completely enough to investigate the surface adsorption [24].

The most stable structure is relatively easy to exist or synthesize, the S1 ~ S3 were designed to measure structures of metal doping on B surface (Fig. 5b). It is found that the structures of S1 and S3 are more stable under static optimization (0 K) than that of S2 [*E*_S1_(0 K) = − 473.69 eV, *E*_S2_(0 K) = − 471.83 eV and *E*_S3_(0 K) = − 474.37 eV]. Interestingly, the stability of S3 is weaker than that of S1 in the AIMD process of structure optimization under the condition of 500 K, corresponding to the average energy of the last 100 fs (inset of Fig. 5b): *E*_S1_ (500 K) = − 475.09 eV vs. *E*_S3_ (500 K) = − 474.44 eV. Therefore, the most stable structure S4 (*E*_S4_(0 K) = − 474.78 eV) was successfully discovered at 8844 fs in the dynamic optimization process of the S1 structure. Furthermore, the structures of Pt@B(104) and the surface oxidation of B_*x*_O(104) and M@B_*x*_O(104) are also tested (Figs. S12 and S13).

For HER (2H^+^  + 2e^−^ → H_2_), we calculated Gibbs free energy of a reaction by the adopting Volmer−Tafel reaction mechanism:

2 $${\text{H}}^{ + } + {\text{ e}}^{ - } \to {\text{H}}^{*}$$

3 $${\text{H}}^{*} + {\text{ H}}^{*} \to {\text{ H}}_{{2}}$$

The Gibbs free energy (ΔG_H*_) for atomic hydrogen (H) adsorption on a catalyst surface has been successfully employed as a descriptor for correlating theoretical predictions [41]. When the value of ΔG_H*_ is close to 0, the reaction rate of both H adsorption and H_2_ desorption reached the maximum. Therefore, seven (B1–B5, M1 and O1) sites were considered to search for the active center of hydrogen evolution (Figs. S14-S16, Table S2). The optimized results (inset in Fig. 5a) shown that only B1–B4 sites can be H adsorption or reaction, so only these sites were further considered. In Fig. 5c, the B1 and O–B1 sites of B(104) and O–B4 sites of Rh@B_*x*_O(104) are an exothermic reaction in process of H^+^  + e^−^ → H^*^ due to the weak interaction with H^*^, whereas all other surfaces sites undergo the endothermic process. Noting, it is difficult for H^*^ to desorb from these sites of B2 and B3 at room temperature (*E*_ads_ < − 1.0 eV) due to their extra-strong adsorption ability, which results in catalyst poisoning and is impossible to complete the catalytic cycle. Therefore, the surface of B4 is the active site of the reaction for the Rh@B_*x*_O(104) surface. Compare with the Pt@B(104), the barrier of HER was obvious to reduce after Rh was compounded with B(104) surface. However, Pt@B(104) exhibited poor catalytic activity due to the exceed adsorption of hydrogen. In addition, oxidated B(104) surface plays a vital role to enhance the hydrogen evolution activity on B4 site of Rh@B_*x*_O(104) (from − 0.26 to 0.01 eV) and O–B1 site for Pt@B_*x*_O(104) (from − 0.57 to − 0.18 eV). Moreover, the performance of Rh@B_*x*_O(104) was more excellent than those of Pt NPs(111) surface (− 0.08 eV) and Rh NPs (111) surface (− 0.18 eV).

To investigate the interaction of the metal and B support, a single-layer B_32_ sheet model, containing hexagonal hollow, is a highly stable structure, which has been proved by previous calculations and experimental evidence [24, 42]. The BE was calculated as follows: BE = *E*_slab+metal_ − *E*_slab_ − *E*_metal_. The average BE value (− 2.983 eV/atom) is lower than that of Rh@B_32_ (− 4.11 eV/atom), which demonstrated strong selectivity between Rh and B support (Fig. 5d and Table S3). The charge density difference also clearly displays that the introduction of electronegativity O can strengthen the Rh-B interaction, with the corresponding BE of Rh@B and Rh@B_*x*_O(104) is − 5.36 eV/atom and − 7.17 eV/atom, which is consistent with the result of Pt and B (Fig. S17). Overall, based on a stable surface, the SMSI effect has been further confirmed, the oxidized B surface was helpful to couple with the metal and endow Rh@B(104) superior HER performance.

## Conclusions

In summary, we have successfully synthesized 2D ultra-thin BNS through a novel sonication-assisted liquid-phase exfoliation method. By choosing NMP and Et-OH (1:1 v/v) as exfoliating solvents, B sheets with a thickness of about 4–8 nm and a lateral size of about 150–250 nm can be prepared in a controlled manner. Additionally, we designed ultra-small Rh NP (average diameter only 3 nm) firmly anchored in 2D ultra-thin BNS nanomaterials for the first time via a rapid NaBH_4_ reduction and facile freeze-dry approach. The Rh NP@BNS possesses plenty of attractive and exciting discoveries: (1) a highly dispersed Rh NP catalyst with a lateral size of only 3 nm can be achieved with a high Rh loading of up to 1.11 wt%; (2) the thin layer of B_2_O_3_ derived by support encapsulates the Rh NP fixed on the BNS, which is the classic SMSI effect. Under electrolysis conditions, Rh is firmly integrated into the BNS framework, which is conducive to electron transfer; (3) the optimized electrocatalyst has excellent HER catalytic activity and stability in the wide pH range electrolytes; (4) the simulated seawater electrolyte still has excellent catalytic activity and durability, highlighting its potential for seawater splitting in some urgent situation; (5) theoretical calculations unraveled that the structure–activity relationship between B(104) crystal plane and Rh metal is beneficial to the release of hydrogen, which is consistent with our experimental results. Based on the stable surface, the oxidized B surface facilitates the coupling of BNS and metal, giving catalyst outstanding HER performance. The current work provides a pioneering strategy for the green preparation of future 2D electrocatalytic HER materials. By accurately designing the SMSI effect, it may open a new path for the development and operation of SMCs with high catalytic activity.

## Supplementary Information

Below is the link to the electronic supplementary material.

Supplementary file1 (DOCX 21545 kb)
