# Supplementary material for: Boron Nanosheet-Supported Rh Catalysts for Hydrogen Evolution: A New Territory for the Strong Metal-Support Interaction Effect
Source: Nanomicro Lett. 2021 Jun 8;13:138. doi: 10.1007/s40820-021-00662-y (PMC8187687; doi:10.1007/s40820-021-00662-y)
Supplement: Supplementary file 1 — Supplementary file1 (DOCX 21545 kb) [file 40820_2021_662_MOESM1_ESM.docx]

Supporting Information for

**Boron Nanosheet-Supported Rh Catalysts for Hydrogen Evolution: A New Territory for the Strong Metal-Support Interaction Effect**

Keng Chen,^†,¶^ Zeming Wang,^†,¶^ Liang Wang,^*,†^ Xiuzhen Wu,^†^ Bingjie Hu,^†^ Zheng Liu,^*,§^ and Minghong Wu^*,‡,‖^

^†^Institute of Nanochemistry and Nanobiology, School of Environmental and Chemical Engineering, Shanghai University, 99 Shangda Road, BaoShan District, Shanghai 200444, P.R. China

^§^School of Materials Science and Engineering, Nanyang Technological University, 50 Nanyang Avenue, Singapore 639798, Singapore

^‡^Shanghai Applied Radiation Institute, Shanghai University, 333 Nanchen Road, Baoshan District, Shanghai 200444, P.R. China

^‖^Key Laboratory of Organic Compound Pollution Control Engineering (MOE), Shanghai University, Shanghai 200444, P. R. China

^¶^These authors contributed equally to this work.

*E-mail: wangl@shu.edu.cn (L. Wang); Z.Liu@ntu.edu.sg (Z. Liu); mhwu@shu.edu.cn (M. Wu).

**Fig. S1** **a**, **b** Three-dimensional AFM images of BNS and Rh NP@BNS. **c**, **d** The corresponding height profile for BNS. **e** The corresponding height profile for Rh NP@BNS.

**Fig. S2** **a** TEM images of Bulk B. **b** High-resolution TEM images of Rh NP@BNS (inset: lateral size distribution of Rh NP).

**Fig. S3** SEM images of **a** Bulk B, **b** BNS, **c** Rh NP@BNS.

**Fig. S4 a** XRD patterns of Bulk B and BNS, Matching PDF card (JCPDS No. 71-0157). **b** XRD patterns of Rh NP@BNS, Compare with B_2_O_3_ PDF card (JCPDS No. 06-0297). **c** The enlarged part of the XRD pattern of Rh NP @ BNS from 30° to 90°, and the corresponding Rh PDF card (JCPDS No. 88-2334).

**Fig. S5** Typical SEM image of Bulk B, corresponding elemental mapping images of B, C, N and O.

**Fig.** **S6** Typical SEM image of BNS, corresponding elemental mapping images of B, C, N and O.

**Fig. S7** Typical SEM image of Rh NP@BNS, corresponding elemental mapping images of B, C, N, O and Rh.

**Fig. S8** HER polarization curves of x-Rh NP@BNS (x=0, 0.5, 1.0, 2.0, 3.0, 5.0, 7.0, x represents the mass percentage of Rh added during the preparation process) performed in 0.5 M H_2_SO_4_ electrolyte.

**Fig. S9** HER polarization curves of x-Rh NP@BNS (x=0, 0.5, 1.0, 2.0, 3.0, 5.0, 7.0, x represents the mass percentage of Rh added during the preparation process) performed in 1.0 M KOH electrolyte.

**Fig. S10** **a** HER LSV curves of Rh NP@BNS performed in 1.0 M NaCl, 1.0 M NaCl+0.5 M H_2_SO_4_, and 1.0 M NaCl+1.0 M KOH electrolyte. **b** Comparison of the overpotentials at 10 mA cm^2^.

**Fig. S11.** Time dependence of current density at 489 mV versus RHE in 1.0 M NaCl electrolyte.

**Fig. S12** Static optimization structure of Pt@B(104).

**Fig. S13** Static optimization of surface oxidation structures of B*_x_*O(104), Rh@B*_x_*O(104) and Pt@B*_x_*O(104).

**Fig. S14** Geometries Structures for the H adsorption of B(104) and B*_x_*O(104) Surface.

**Fig. S15** Geometries Structures for the H adsorption of Rh@B(104) and Rh@B*_x_*O(104) Surface.

**Fig. S****16** Geometries Structures for the H adsorption of Pt@B(104) and Pt@B*_x_*O(104) Surface.

**Fig. S17** Isosurface of charge density difference (Δ*ρ*) for Rh@B*_x_*O(104), Pt@B*_x_*O(104).

**Table S1** The total Rh content of the deposition was obtained by ICP-oes test.

| Element | Weight/g | Volume/mL | Dilution  factor | Instrument  Readings mg/L | Concentration  mg/kg | Percentage wt% |
| --- | --- | --- | --- | --- | --- | --- |
| Rh | 0.0244 | 50 | 1 | 5.4157 | 11097.6876 | 1.11 |

**Table S2** DFT-calculated ΔGH* of various Geometries Structures for the H adsorption of B(104), B*_x_*O(104), M@B(104) and M@B*_x_*O(104) Surface (M=Rh, Pt).

| **Doping structures** | **Sites** | **E_DFT_** | **Ox** | **Sites** | **E_DFT_** |
| --- | --- | --- | --- | --- | --- |
|  | 1 | **-470.91** |  | 1 | **-478.30** |
| B(104) | 2 | -472.49 | B_x_O(104) | 2 | -478.97 |
|  | 3 | -472.64 |  | 3 | -480.22 |
|  | 1 | **-478.56** |  | 1 | -487.85 |
|  | 2 | -469.99 |  | 2 | -487.02 |
|  | 3 | -478.56 |  | 3 | -487.81 |
| Rh@B(104) | 4 | -471.27 | Rh@B*_x_*O(104) | 4 | -487.95 |
|  | 5 | -479.60 |  | 5 | -489.00 |
|  | 6 | - |  | 6 | - |
|  | - | - |  | 7 | -487.29 |
|  | 1 | **-478.22** |  | 1 | **-487.39** |
|  | 2 | - |  | 2 | - |
|  | 3 | -477.44 |  | 3 | -486.81 |
| Pt@B(104) | 4 | -477.65 | Pt@B*_x_*O(104) | 4 | - |
|  | 5 | -479.19 |  | 5 | -488.84 |
|  | 6 | - |  | 6 | - |
|  | - | - |  | 7 | -486.81 |

**Table S3** DFT-calculated binding energy (BE) of various B_32_ doping structures, spin magnetic moment (μ) of single metal atoms were also listed.

| **Doping structures** | **BE(eV)** | **μ(μ_B_)** |
| --- | --- | --- |
| Ti | -3.45 | 4.00 |
| Mn | -1.98 | 5.00 |
| Fe | -3.22 | 4.00 |
| Ni | -3.85 | 2.00 |
| Cu | -2.03 | 1.00 |
| Mo | -2.98 | 6.00 |
| Pt | -3.49 | 2.00 |
| Rh | -4.17 | 1.00 |
| Pd | -1.68 | 0.00 |

**Table S4** Comparison of HER activities of catalysts in acidic electrolytes (0.5 M H_2_SO_4_)

| **Catalyst** | **Rh metal content (wt%)** | **Rh nanoparticle size (nm)** | **Overpotential at**  **10 mA cm^-2^ (mV)** | **Tafel slope**  **(mV dec^-1^)** | **Reference** |
| --- | --- | --- | --- | --- | --- |
| Rh NP@BNS | 1.11 | ~3 | 66 | 56 | This work |
| Rh-Ag/SiNW | 2.3 | 12.3 | 120 | 51 | [S1] |
| Rh-Au/SiNW | 2.2 | 15.8 | 62 | 24 | [S2] |
| rGO/CoP-Rh | NA | 12.86 | 72 | 43 | [S3] |
| Rh/SWNTs | 6.1 | ~2 | 25 | 20 | [S4] |
| MoSe_2_/Rh | NA | ~8 | 192 | 47 | [S5] |
| Rh/F-graphene | 9.2 | 9.39 | 46 | 30 | [S6] |
| Rh-CN | 4.2 | 3.4 | 13 | 25 | [S7] |
| Rh/Ni@NCNTs | 2.84 | 1.92 | 45 | 37.2 | [S8] |
| B-RhFe alloy | ~18.29 | 4.01 | 25 | 32 | [S9] |
| Rh-Rh_2_P@C | NA | 3.4 | 24 | 35.8 | [S10] |

**Table S5** Comparison of HER activities of catalysts in alkaline electrolytes (1.0 M KOH)

| **Catalyst** | **Rh metal content (wt%)** | **Rh nanoparticle size (nm)** | **Overpotential at**  **10 mA cm^–2^ (mV)** | **Tafel slope**  **(mV dec^-1^)** | **Reference** |
| --- | --- | --- | --- | --- | --- |
| Rh NP@BNS | 1.11 | 3 | 101 | 75 | This work |
| rGO/CoP-Rh | NA | 12.86 | 155 | 101 | [S3] |
| MoSe_2_/Rh | NA | ~8 | 173 | NA | [S5] |
| Rh/SWNTs | 6.1 | ~2 | 25 | 20 | [S4] |
| Rh–MoSe_2_ nanoflowers | 8.2 | 2.5 | 73 | 118 | [S11] |
| Rh-CN | 4.2 | 3.4 | 55 | 44 | [S7] |
| Rh/Ni@NCNTs | 2.84 | 1.92 | 45 | 37.2 | [S8] |
| Rh-Rh_2_P@C | NA | 3.4 | 37 | 32 | [S10] |
| P-Rh/C | 12.57 | 1.98 | 11 | 71.4 | [S12] |
| Rh/N-CBs | 3.5 | ~1.4 | 77 | 74.16 | [S13] |
| Rh NSs | NA | 18.4 | 43 | 107.2 | [S14] |

**Supplementary References**

[S1] B. Jiang, Y. Sun, F. Liao, W. Shen, H. Lin, H. Wang, M. Shao, Rh–Ag–Si ternary composites: highly active hydrogen evolution electrocatalysts over Pt–Ag–Si, J. Mater. Chem. A 5 (2017) 1623-1628.

[S2] B. Jiang, L. Yang, F. Liao, M. Sheng, H. Zhao, H. Lin, M. Shao, A stepwise-designed Rh-Au-Si nanocomposite that surpasses Pt/C hydrogen evolution activity at high overpotentials, Nano Res. 10 (2017) 1749-1755.

[S3] H. Zheng, X. Huang, H. Gao, W. Dong, G. Lu, X. Chen, G. Wang, Decorating cobalt phosphide and rhodium on reduced graphene oxide for high-efficiency hydrogen evolution reaction, J. Energy Chem. 34 (2019) 72-79.

[S4] W.Q. Zhang, X. Zhang, L. Chen, J.Y. Dai, Y. Ding, L.F. Ji, J. Zhao, M. Yan, F.C. Yang, C.R. Chang, S.J. Guo, Single-Walled Carbon Nanotube Induced Optimized Electron Polarization of Rhodium Nanocrystals To Develop an Interface Catalyst for Highly Efficient Electrocatalysis, Acs Catal. 8 (2018) 8092-8099.

[S5] M.D. Sharma, C. Mahala, M. Basu, Nanosheets of MoSe_2_@M (M=Pd and Rh) function as widespread pH tolerable hydrogen evolution catalyst, J. Colloid Interface Sci. 534 (2019) 131-141.

[S6] W. Shen, L. Ge, Y. Sun, F. Liao, L. Xu, Q. Dang, Z. Kang, M. Shao, Rhodium Nanoparticles/F-Doped Graphene Composites as Multifunctional Electrocatalyst Superior to Pt/C for Hydrogen Evolution and Formic Acid Oxidation Reaction, ACS Appl. Mater. Interfaces 10 (2018) 33153-33161.

[S7] B. Jiang, A. Huang, T. Wang, Q. Shao, W. Zhu, F. Liao, Y. Cheng, M. Shao, Rhodium/graphitic-carbon-nitride composite electrocatalyst facilitates efficient hydrogen evolution in acidic and alkaline electrolytes, J. Colloid Interface Sci. 571 (2020) 30-37.

[S8] Q. Wang, B. Xu, C. Xu, Y. Wang, Y. Zhang, J. Wu, G. Fan, Ultrasmall Rh nanoparticles decorated on carbon nanotubes with encapsulated Ni nanoparticles as excellent and pH-universal electrocatalysts for hydrogen evolution reaction, Appl. Surf. Sci. 495 (2019) 143569.

[S9] L. Zhang, J. Lu, S. Yin, L. Luo, S. Jing, A. Brouzgou, J. Chen, P.K. Shen, P. Tsiakaras, One-pot synthesized boron-doped RhFe alloy with enhanced catalytic performance for hydrogen evolution reaction, Appl. Catal. B-Environ. 230 (2018) 58-64.

[S10] F. Luo, L. Guo, Y. Xie, J. Xu, W. Cai, K. Qu, Z. Yang, Robust hydrogen evolution reaction activity catalyzed by ultrasmall Rh–Rh_2_P nanoparticles, J. Mater. Chem. A 8 (2020) 12378-12384.

[S11] Y. Zhao, C. Yang, G. Mao, J. Su, G. Cheng, W. Luo, Ultrafine Rh nanoparticle decorated MoSe_2_ nanoflowers for efficient alkaline hydrogen evolution reaction, Inorg. Chem. Front. 5 (2018) 2978-2984.

[S12] L. Su, Y. Zhao, F. Yang, T. Wu, G. Cheng, W. Luo, Ultrafine phosphorus-doped rhodium for enhanced hydrogen electrocatalysis in alkaline electrolytes, J. Mater. Chem. A 8 (2020) 11923-11927.

[S13] N. Jia, Y. Liu, L. Wang, P. Chen, X. Chen, Z. An, Y. Chen, 0.2 V Electrolysis Voltage-Driven Alkaline Hydrogen Production with Nitrogen-Doped Carbon Nanobowl-Supported Ultrafine Rh Nanoparticles of 1.4 nm, ACS Appl. Mater. Interfaces 11 (2019) 35039-35049.

[S14] N. Zhang, Q. Shao, Y. Pi, J. Guo, X. Huang, Solvent-Mediated Shape Tuning of Well-Defined Rhodium Nanocrystals for Efficient Electrochemical Water Splitting, Chem. Mater. 29 (2017) 5009-5015.
